# Functional Coupling and Evolutionary Relationships Between Toxin–Antitoxin Systems and CRISPR-Cas Systems

**DOI:** 10.3390/toxins17120602

**Published:** 2025-12-16

**Authors:** Yibo Meng, Jiyun Chen, Liang Liu

**Affiliations:** State Key Laboratory of Cellular Stress Biology, School of Life Sciences, Faculty of Medicine and Life Sciences, Xiamen University, Xiamen 361102, China

**Keywords:** CRISPR-Cas system, toxin-antitoxin system, CreTA system, CreR system, CrePA system, AbiF system, Cas13

## Abstract

Bacteria encode a broad range of survival and defence systems, including CRISPR (Clustered Regularly Interspaced Short Palindromic Repeats)-Cas systems, restriction-modification systems, and toxin–antitoxin (TA) systems, which are involved in bacterial regulation and immunity. The traditional view holds that CRISPR-Cas systems and TA systems are two independent defense lines in prokaryotes. However, groundbreaking studies in recent years have revealed multi-level functional coupling between them. This review systematically elaborates on this mechanism, focusing on three types of TA systems that mediate the core correlation of CRISPR-Cas systems: CreTA maintains the evolutionary stability of CRISPR-Cas systems through an addiction mechanism; CreR enables self-regulation of CRISPR-Cas expression; and CrePA provides herd immunity by triggering abortive infection after the CRISPR-Cas system has been destroyed by Anti-CRISPRS protein. Additionally, we discuss the evolutionary homology between the type III toxin AbiF and the type VI CRISPR effector Cas13, offering a new perspective for understanding the origin of CRISPR-Cas systems. These findings not only reveal the functional coupling of prokaryotic defense systems but also provide a powerful theoretical framework and practical solutions for addressing stability challenges in CRISPR technology applications.

## 1. Introduction

Toxin–antitoxin systems and CRISPR-Cas systems are two major classes of genetic elements in bacteria. As a widely existing antiviral immune system in prokaryotic microorganisms, the CRISPR-Cas system recognizes and cleaves invading nucleic acids in a sequence-specific manner, providing heritable immune memory which serves to guard against next invasions by foreign nucleic acids [1]. In contrast, toxin–antitoxin systems mediate cell growth arrest or programmed cell death via the toxin element in response to various environmental stresses. Research shows that the function of TA systems has expanded from the initially discovered plasmid stabilization to phage defense and persister cell formation [2,3,4,5].

Although these two systems are different in terms of their mechanisms and functions. There is an inseparable connection between the two. They do not operate in isolation; instead, they form an internally communicating defense alliance. When external pathogens invade bacteria, the CRISPR-Cas system will be activated first. If the defense fails, the TA system can serve as the ultimate defense line to induce dormancy or death. More notably, newly identified RNA-based Type VIII TA systems directly affect the stability of CRISPR-Cas systems [6]. The discovery of CreTA revealed that CRISPR-Cas loci themselves may be genetic units containing the TA system. Meanwhile, the development of novel antibacterial strategies, Associate toxin–antitoxin and CRISPR-Cas to kill MDR (multidrug resistance) pathogens (ATTACK) [7] and the confirmation that the Type III toxin AbiF is an evolutionary precursor of the Cas13 protein [8] both strongly demonstrate the close connection between these two systems. This review aims to systematically organize the relationships between CRISPR-Cas and TA systems. Through discussing representative TA systems including CreTA, CreR, CrePA and AbiF, we will illustrate how bacteria utilize the coupling of TA systems and CRISPR-Cas to overcome challenges of foreign invasion, and the evolutionary origin of the CRISPR system.

## 2. Introduction of the Two Systems

### 2.1. Classification and Functions of TA Systems

**Figure 1:**
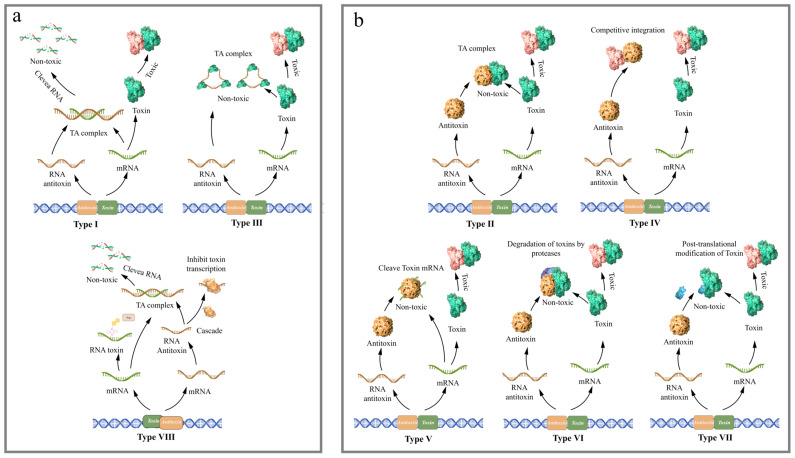
The classification and the neutralization mechanisms of TA system. (**a**) In type I, III, and VIII TA systems, antitoxins are RNAs, toxins are proteins, with the exception of type VIII toxins (toxins are RNAs). (**b**) The type II, IV, V, VI, and VII TA system toxins and antitoxins are proteins. (By Figdraw.).

Toxin–antitoxin systems are small genetic modules widely distributed in prokaryotic genomes. They were initially discovered to play a role in plasmid maintenance. They generally are located on plasmids or other mobile genetic elements, enabling them to transfer between organisms via horizontal gene transfer. The first characterized TA system was a plasmid-borne Type II system, which functions in plasmid maintenance through a mechanism called post-segregation killing [9,10]. When a plasmid encoding a TA system is lost from a cell, the inability to produce new antitoxins allows residual stable toxins to persist, ultimately leading to cell death. As continuous exploration, more TA systems were also discovered on chromosomes [11,12,13,14]. TA systems are classified into eight types based on functional analyses of toxin and antitoxin activities. The toxins of all known TA systems are proteins, with the exception of type VIII toxins (RNAs); the antitoxins are RNA or protein. In type I, III, and VIII TA systems, antitoxins are RNAs. The type II, IV, V, VI, and VII TA system toxins and antitoxins are proteins (Figure 1). In type I TA systems, the toxins are hydrophobic short peptides that disrupt bacterial membrane integrity by causing depolarization or altering permeability, thereby impairing membrane potential and cell division. The antitoxins are small RNAs that bind to toxin mRNAs to promote their degradation, block toxin translation, and inhibit the transcription of their cognate toxins [15,16,17,18,19,20,21]. In Type II TA systems, both the toxins and antitoxins are proteins. They directly bind to form a complex that neutralizes toxin activity. Both the antitoxin alone and the toxin–antitoxin complex can bind to the promoter region of the TA operon, enabling self-regulation [22,23,24,25,26]. In Type III TA systems, the toxins are typically endonucleases, and the antitoxins are sRNAs. The antitoxins bind to their cognate toxins and neutralize the toxin proteins by forming protein-RNA complexes [27,28,29,30]. In Type IV TA systems, both the toxins and antitoxins are proteins that share the same target but do not bind to each other. Antitoxins inhibit toxin activity by competitively binding to the substrate of the toxin [31,32]. In Type V TA systems, the antitoxins are ribonucleases that degrade toxin mRNAs to inhibit toxin expression. Toxins induce host growth arrest by disrupting cell membranes, while antitoxins suppress toxicity by targeting and cleaving toxin mRNAs [33,34,35]. In Type VI TA systems, the antitoxins act as adaptor proteins to target toxins to proteases. Toxins inhibit cell activity by blocking the elongation of DNA replication, whereas antitoxins promote toxin degradation by proteases [36,37]. In Type VII TA systems, the antitoxins are enzymes that inactivate toxins through post-translational modifications, such as phosphorylation and adenylylation [38,39]. In Type VIII TA systems, both the toxins and antitoxins are RNAs. Toxins primarily function by blocking tRNAs or inhibiting mRNA targets, while antitoxin RNAs directly degrade toxin RNAs and recruit Cas proteins to act as transcriptional repressors [40]. The diversity of the TA systems reflects their evolutionary success and functional plasticity.

### 2.2. Classification and Tool-Based Applications of CRISPR-Cas Systems

The CRISPR-Cas system is an adaptive defence system widely present in bacteria and archaea, serving to defend against the invasion of exogenous nucleic acids. Through engineering modification and design, this system has become a highly efficient gene-editing tool and is extensively applied in basic biology [41,42]. With the deepening of research, new types of CRISPR-Cas systems are constantly being discovered; currently, dozens of subtypes of CRISPR-Cas systems have been identified [43,44]. The classification of CRISPR-Cas systems is mainly based on the composition and function of effector complexes, as well as the conservation of *cas* genes, characteristics of repeat sequences, and target types (DNA or RNA). Based on these criteria, CRISPR-Cas systems are divided into two major categories and multiple subtypes [45]. Among them, Cas9 from Type II systems; Cas12a from Type V systems and Cas13a from Type VI systems (Table 1), due to their simple single-protein effector structure, have been successfully developed into revolutionary genome-editing tools. They are widely used in fields such as gene knockout, gene screening, gene therapy, antibacterial agents and crop breeding [46,47,48,49,50,51,52,53,54,55,56]. However, as a tool, CRISPR-Cas systems currently have certain limitations, including low editing efficiency, potential off-target editing risks, and restricted editing accessibility due to protospacer adjacent motif requirements. Addressing these issues requires further in-depth research.

## 3. A Defense Network with Functional Coupling: Mechanisms and Applications

### 3.1. CreTA-The Guardians of the CRISPR-Cas Genome

The CreTA system is a Type VIII TA system nested within the CRISPR-Cas locus. This system was discovered in *Haloarcula hispanica*. Toxin CreT is not a typical toxin protein; its mRNA itself exhibits toxicity, the toxic mechanism relying on two key features: first, it possesses an unusually strong ribosome-binding site that efficiently hijacks the translation initiation complex [57,58,59,60,61,62,63]; second, its open reading frame contains rare AGA codons, which extensively sequester and deplete the already scarce tRNA ^UCU^ in cells. This severe disruption of host protein synthesis ultimately leads to cell growth arrest [64]. Similarly, antitoxin CreA is a crRNA (CRISPR RNA)-like molecule that needs to be processed by the Cas6 enzyme to become mature. The mature CreA binds to the Cascade complex in the type I-B CRISPR-Cas system. Due to the partial complementarity between the spacer sequence of CreA and the promoter region P*creT*, the Cascade-CreA complex binds to P*creT* through its complementary parts, physically repressing the transcription of creT through transcriptional interference, thereby achieving detoxification [65]. The mechanism of CreTA enables it to function as a “genomic guardian” of the CRISPR-Cas system. While CRISPR-Cas provides prokaryotes with adaptive immunity inside the bacteria, it also imposes costs on the host, such as autoimmunity due to spacer mismatches or accidental targeting of essential symbiotic plasmids [66,67,68]. These burdens may confer a growth advantage to strains that lose the CRISPR-Cas system in environments without persistent phage pressure. However, CreTA addresses this evolutionary instability through an enforced “addiction” mechanism [69,70,71]. Any mutation that disrupts Cascade function—such as deletion of *cas6* or *cas8*—prevents maturation or performs normal functions of CreA, thereby lifting suppression of the CreT toxin and leading to growth arrest. This effectively exerts a suppressive effect on hosts that attempt to abandon the defense system and ensures vertical inheritance of the CRISPR-Cas locus.

Although CreTA differs from classical TA systems in many aspects, it still represents a highly atypical TA system. Conventional TA system toxins are predominantly proteins that disrupt critical cellular processes through enzymatic hydrolysis. However, the toxicity of CreT stems from its mRNA molecule itself, which is a strategy characterized by exceptional economy and efficiency. This involves hijacking translation initiation via an ultra-strong ribosome-binding site and depleting the rare tRNA ^UCU^, synergistically amplifying its inhibitory effect. Correspondingly, the antitoxin CreA is a crRNA-like molecule that requires processing by the Cas6 enzyme. This represents a functional linkage to the CRISPR-Cas system and the expansion of CRISPR-Cas system functionality. From cutting to regulation, the traditional function of Cas proteins and the Cascade complex is to recognize and cut exogenous nucleic acids. However, in the association of the CRISPR-CreTA system, the same Cas protein complex is recruited to perform a completely new task, which is to regulate the activity of an endogenous toxin RNA. Simultaneously, the discovery of CreTA expands the list of elements in the CRISPR-Cas cassette and extends the known functions of Cas proteins in shaping the genomes and transcriptomes of diverse prokaryotes [72]. That is a significant advance in the functional study of CRISPR-Cas systems. Meanwhile, the presence of CreTA within the CRISPR-Cas locus suggests that a complete, heritable system may comprise not only core Cas genes and spacers, but also ancillary elements such as CreTA. Consequently, the definition of a “CRISPR-Cas locus” is redefined as a more complex and integrated genetic module.

### 3.2. CreR-Regulators of the CRISPR-Cas System

CreR is a crRNA-like regulatory RNA widely distributed in Type I and Type V-A CRISPR-Cas systems. Different from CreA, it is generally not coupled with toxin-encoding genes. The core mechanism of CreR lies in its ability to form regulatory complexes with Cas proteins, such as the Cascade complex in Type I systems or Cas12a in Type V-A systems. This complex can inhibit the expression of Cas proteins by specifically recognizing and binding to the promoter region of its cognate *cas* operon [73]. Thereby achieving CRISPR-Cas autoregulation. The CreR system implements an elaborate strategy for optimizing physiological costs in prokaryotes. Upon phage invasion, a large number of exogenous spacers are transcribed and processed into crRNAs. These immune crRNAs compete with the regulatory CreR for binding to the limited pool of Cas proteins within the cell. Owing to the higher affinity of crRNAs for Cas proteins or their inherent binding priority, Cas proteins are extensively recruited to assemble immune effector complexes. This recruitment leads to a reduction in the abundance of CreR-Cas inhibitory complexes, which in turn automatically relieves the transcriptional repression of *cas* genes. Consequently, the *cas* gene expression is upregulated to meet the increased demand for antiviral defense. After the phage threat is eliminated, the level of immune crRNAs declines. At this stage, CreR rebinds to Cas proteins, reforming CreR-Cas inhibitory complexes and re-suppressing Cas expression to conserve cellular resources [74]. To protect themselves from CRISPR-Cas systems, bacteriophages produce inhibitory Acr (anti-CRISPR) proteins. Acr proteins inhibit CRISPR-Cas system activity by disassembling the Cascade [75,76,77,78]. Following the invasion and neutralization of a subset of Cas proteins by Acr proteins, the stability of the CreR-Cas repression complex is indirectly compromised, thereby de-repressing Cas genes. The subsequent increase in Cas protein expression may then titrate out the inhibitory capacity of Acr proteins, constituting a transcriptional ‘anti-Anti-CRISPR’ strategy.

The discovery of the CreR system further validates the interconnectedness of prokaryotic defence systems. Unlike the CreTA system, which compels the host to retain the core functions of the entire CRISPR-Cas system, the purpose of CreR is to achieve self-regulation of the CRISPR-Cas system within the cell. It finely modulates the expression levels of the host’s own Cas proteins through transcriptional repression, avoiding resource waste and potential autoimmune risks caused by excessive expression. This advances the concept of CRISPR-Cas defense systems from a static “immune gene repository” to a new paradigm of a dynamic “resource management system.” The integration of the CreR system with the CRISPR-Cas system establishes a rapid-response switch centered on “molecular competition.” Traditional transcriptional regulation typically relies on the expression, modification of protein transcription factors, or their binding to small molecules. The inherent time costs of transcription and translation limit its response speed. The revolutionary nature of the CreR system lies in its ability to directly couple regulatory signals in the form of RNA with regulatory execution at the level of protein complex assembly. When phage DNA is cleaved to generate large quantities of crRNA, these crRNAs do not require any signal transduction pathways. Instead, they act directly as “molecular sponges” to “compete” for binding partners within the existing intracellular pool of Cas proteins. This competition, governed by the law of mass action, is a physicochemical process with an extremely rapid response speed—repression can be relieved in as little as seconds or minutes, buying valuable defense time for the host.

Furthermore, the activation signal of this system is a high-abundance crRNA derived from exogenous DNA. This ensures that *cas* genes are only massively activated when a genuine invasion (recognizable by the CRISPR system) occurs, avoiding false activation and resource waste under harmless stimuli. Sustained expression of large cas operons imposes a heavy burden on cellular energy and material resources. In environments lacking persistent phage pressure, strains with constitutively high CRISPR system expression will be at a disadvantage in growth competition. The CreR system perfectly resolves this “tragedy of the commons” (the overall disadvantage caused by the excessive consumption of public resources by individuals in a group) by establishing an infrastructure management strategy of “activation on demand, shutdown when idle.” This strategy forces phages to confront new evolutionary challenges.

### 3.3. CrePA-Defenders of Herd Immunity

**Figure 2:**
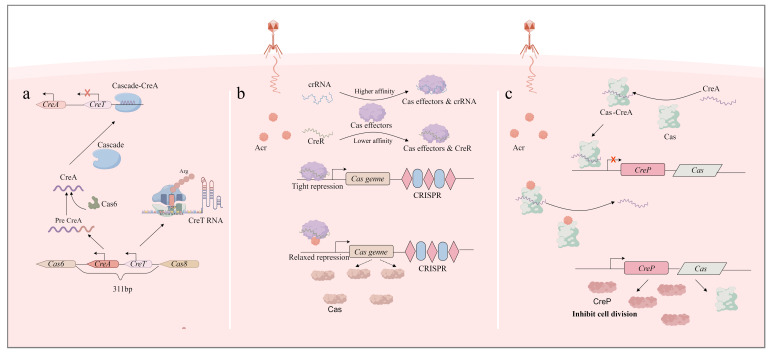
Toxin–antitoxin CreTA; CreR; CrePA: (**a**) Toxin–antitoxin RNA pair CreTA safeguards CRISPR-Cas. CRISPR effector (Cascade) is opted by CreA RNA to transcriptionally repress the toxin gene creT through partial complementarity between CreA and the creT promoter. When Cascade is inactivated, the derepressed CreT RNA sequesters the rare tRNA^UCU^ that decodes a minor arginine codon and arrests cellular growth, thus making the CRISPR effector addictive to the host cell. (**b**) CreR regulates the expression of Cas genes by sensing the concentration of crRNA and the presence of Acr protein. The CreR system implements a sophisticated strategy for optimizing the physiological costs in prokaryotes. Upon phage invasion, a large number of exogenous DNA spacers are transcribed and processed into crRNAs. These immune crRNAs compete with the regulatory CreR for binding to a small pool of Cas proteins present. Due to a higher affinity of crRNAs for Cas proteins they are extensively recruited to assemble immune effector complexes. This recruitment then leads to a reduction in the abundance of CreR-Cas inhibitory complexes that relieve the transcriptional repression of cas genes. Once the phage threat is kept under control, the levels of immune crRNAs decline. CreR rebinds Cas proteins, reforming CreR-Cas inhibitory complexes and suppressing expression of Cas. (**c**) CreP induces cell death by sensing the specific repression of CRISPR by Acr. CreA binds with the Cas effector and inhibits the expression of the CreP gene. When anti-CRISPR proteins produced during phage invasion cause the Cas-CreA complex to disassemble, CreP protein is expressed and exerts a toxic effect. (By Figdraw.).

Bacteria utilize small CRISPR RNAs and Cas proteins to efficiently combat various mobile genetic elements, such as phages and plasmids; in response, phages produce Acr proteins to protect themselves [79,80,81,82,83,84]. To date, over 100 families of Acr proteins encoded by mobile genetic elements (MGEs) have been identified, with most known to inhibit Type I CRISPR systems [85,86]. The toxin–antitoxin module can be activated by phage-encoded Acr proteins or Racr RNAs [87,88], triggering abortive infection that prevents phage propagation within the bacterial population [89]. The CrePA system represents a more advanced form of coupled defense. Its toxin, CreP, is a protein that typically contains a phage-derived KilA-N domain; it prevents bacterial cell division by interfering with the cell division protein FtsZ, leading to cell filamentation [90,91,92,93,94]. Its antitoxin, CreA, is also a crRNA-like molecule. Under normal conditions, CRISPR-Cas effector complexes bind to the promoter region of the creP gene under the guidance of CreA, repressing creP transcription and avoiding self-toxicity to the bacterium. This repression depends on the normal function of CRISPR-Cas effectors and serves as the basis for the synergistic interaction between the two systems. When phages carrying Acr proteins invade, the function of CRISPR-Cas effectors is disrupted, which relieves the transcriptional repression of creP by the Cascade-CreA complex and activates creP expression. Expressed CreP inhibits cell division and induces cell filamentation, ultimately preventing the proliferation of Acr-infected cells. Phages or plasmids carrying Acr proteins are eliminated along with the death of the host cell, while uninfected bacteria survive by retaining intact CRISPR-Cas function. The activated toxicity of CreP produces an effect similar to “abortive infection,” which blocks the spread of Acr-carrying phages within bacterial populations. This effect does not rely on the direct targeting of phages by CRISPR-Cas; instead, it protects uninfected cells from the threat of Acr-encoding genetic invaders by eliminating cells disrupted by Acr proteins (Figure 2).

Unlike the traditional CRISPR-Cas immunity that only provides individual-level protection, the addition of the CrePA system raises the defense level to the group level through the “altruistic suicide” mechanism. The association of the CrePA system with the CRISPR system provides bacterial defense with dual safeguards and high specificity. Under uninfected conditions, the CRISPR-Cas effector complex, guided by the antitoxin CreA, continuously suppresses the expression of the toxin CreP, ensuring the safety of the host bacterium. The response of CreP is only triggered when CRISPR is specifically inhibited by Acr proteins, thereby inducing cell death before phage replication spreads through the surrounding bacterial population and preventing population-wide infection. In contrast, the inactivation resulting from intrinsic mutations or loss of the CRISPR system itself does not induce toxin expression, thus preventing self-damage. This mechanism constitutes a highly specific ‘anti-Acr’ defense system. It provides a new view for understanding how prokaryotes optimize the allocation of immune resources and associate the TA and CRISPR-Cas systems to cope with highly specific threats. The phages dismantle CRISPR-Cas defenses via Acr proteins, leading to the host being destroyed and subsequent infection of the bacterial population. However, the ability of the CrePA system prevents the spread of bacteriophages within the bacterial population by inducing the premature death of Acr-carrying bacteria before bacteriophages can complete reproduction. This mechanism not only eliminates the bacteriophages’ infectious threat but, more importantly, actively cleans up individuals carrying Acr elements from the population. Consequently, it creates a survival advantage for the bacterial population, ultimately establishing a form of “herd immunity.” Through the CrePA system, the pathogen’s escape mechanism is repurposed as a trigger to launch a more robust defensive response.

Studies on these three toxin–antitoxin systems also reveal that the CRISPR-Cas system is by no means a simple binary regulatory system, but rather a sophisticated regulatory network that collaborates with other defense systems. CreTA serves as a guardian to prevent the loss of the CRISPR-Cas system and address issues related to long-term evolutionary stability. CreR functions as a regulator to regulate CRISPR-Cas system expression levels and resolve problems of short-term physiological cost optimization. The signaling molecule crRNA acts as a key indicator, dynamically adjusting the “regulator” and responding to external threats. CrePA specifically counters Acr predators that have destroyed CRISPR-Cas system capabilities (Table 2). Each of these mechanisms provides its host with a critical survival advantage in specific ecological niches. This cognitive framework not only deepens our understanding of the diversity and environmental adaptability of prokaryotic defence strategies but also provides theoretical support and modular technical tools for developing tailored solutions in synthetic biology.

## 4. Evolutionary Relationships Between CRISPR-Cas Systems and TA Systems

**Figure 3:**
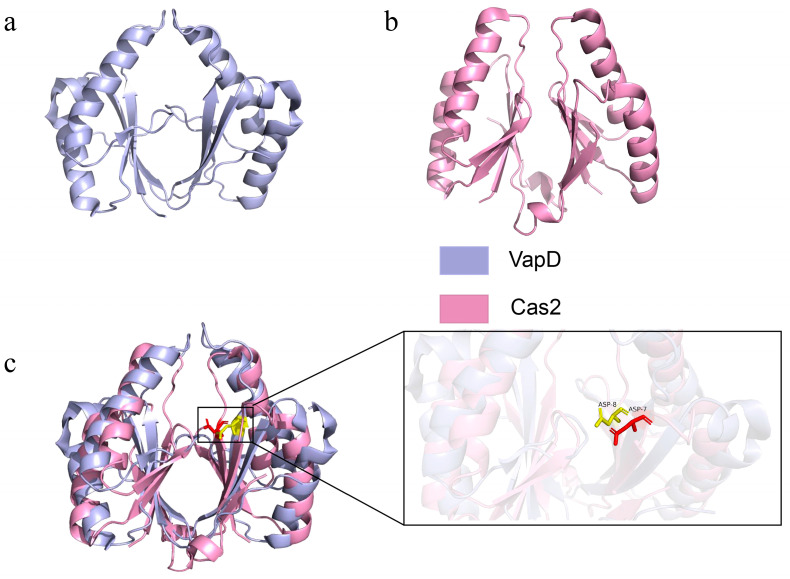
Structural diagrams of VapD (6ZI0) and Cas2 (4QR0). (**a**) Structural diagrams of VapD. (**b**) Structural diagrams of Cas2. (**c**) Structural superposition of VapD and Cas2 based on their dimer structures. The purple VapD and the pink Cas2 dimers are shown, with the conserved acidic residues (Asp7 in VapD and Asp8 in, respectively) highlighted in the box.

Apart from functional coupling, CRISPR-Cas systems and TA systems even share evolutionary origins. Evolutionary tracing studies on key proteins such as Cas2 and Cas13 reveal that they most likely originated directly from toxin components of TA systems. Cas2 is a key component of CRISPR-Cas systems; it forms an adaptation complex with Cas1 and is responsible for integrating exogenous DNA fragments into CRISPR arrays [95,96,97,98,99]. Surprisingly, the Cas2 proteins encoded by most CRISPR-Cas adaptive defence systems are distantly homologous to VapD proteins [100]. Structural biology studies show that VapD-like toxin proteins in Type II TA systems have a modified ferredoxin-like fold, closely resembling that of the Cas2 protein family, and, like Cas2 proteins (Figure 3), VapD-like toxin proteins display an intrinsic RNase activity.

Meanwhile, the active site aspartic acid residues in VapD correspond structurally to either aspartic or glutamic acid residues in different Cas2 proteins that coordinate divalent metal ions, indicating their common nuclease ancestor origin. These highly similar structural characteristics strongly suggest that VapD and Cas2 derive from a common ancestral protein. However, over the course of evolution, their functions have diverged significantly: VapD acts as a toxin, inhibiting cell growth through its nuclease activity. In contrast, after being “recruited” to the CRISPR system, Cas2 retains its structural scaffold and enzymatic potential, but its main function may have shifted to that of a scaffold protein. As the structural core of the Cas1-Cas2 integration complex, it is specifically responsible for spacer acquisition [101]. This “structural conservation and functional innovation” is a typical feature of molecular evolution, proving that TA systems are an important evolutionary source of CRISPR system components.

**Figure 4:**
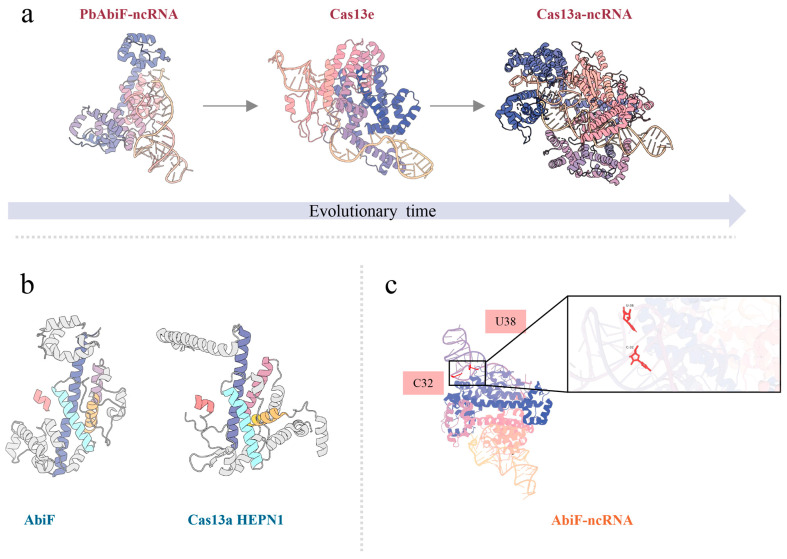
Structure of the PbAbiF-ncRNA; Cas13e; Cas13a-ncRNA; AbiF and Cas13a HEPN1. (**a**) Structure of the PbAbiF-ncRNA (8VZ6); Cas13e (AF3); Cas13a-ncRNA (5XWY). Cas13e is a potential evolutionary intermediate in the evolution from AbiF to Cas13a. (**b**) Both the HEPN domains of AbiF and Cas13a adopt the typical four-helix fold. (**c**) The specific interaction between the PbAbiF protein and the non-coding RNA (ncRNA) called PbAbiFr. Nucleotides C32 and U38 within a specific single-stranded bulge of AbiFr inhibit the non-specific RNase activity of AbiF.

The investigation of the evolutionary origin of type VI CRISPR effector Cas13 provides a clearer picture of the evolutionary path from TA toxin to CRISPR-Cas system, unlike the previously discovered type II CRISPR-Cas9 and type V CRISPR-Cas12, which evolved from RNA-guided nucleases OMEGA-IscB and OMEGA-TnpB related to transposons. The origin of Cas13 has long remained elusive because of the high sequence and structural divergence with the HEPN (Higher Eukaryotes and Prokaryotes Nucleotide-binding) superfamily proteins [102,103,104,105,106]. In the previous investigations, using a traditional sequence alignment, it was difficult to capture any evolutionary association. To tackle this problem, the researchers employed an innovative “structural-sequence hybrid homology search strategy” to conduct large-scale mining of metagenomic databases [107,108,109,110,111,112]. This effort led to the pivotal identification that Cas13 shares an evolutionary branch with AbiF toxin within the Type III TA system, suggesting AbiF as their common ancestor. The phylogenetic analysis suggests the following paths of evolution: AbiF → F13a1/F13a2 → c13c1 (Cas13e) → Cas13a/b/c/d [8]. In the subsequent exploration, the dual characteristics of Cas13e confirm its status as an evolutionary intermediate. It retains the regulatory logic of the “toxin–antitoxin” system from its ancestral TA systems, while simultaneously developing the “RNA-guided targeting” function unique to CRISPR systems. This represents a key transitional form in the evolution from TA systems to CRISPR systems. The explanation for this functional transformation lies in the fact that its underlying driving factor is the co-evolution of non-coding RNA (ncRNA). The antitoxic regulation of AbiF depends on a specific cis-encoded ncRNA named AbiFr. AbiFr functions as the antitoxin by forming a stable ribonucleoprotein complex with AbiF. The AbiF toxin exhibits single-stranded RNA-specific nuclease activity. It not only mediates the cleavage and processing of its cognate ncRNA AbiFr but also displays non-specific ssRNA cleavage activity. During the evolutionary transition from AbiF to Cas13a, the presence of ncRNA AbiFr established the molecular foundation for the subsequent functional specialization of crRNAs, particularly in mediating targeted recognition. The crRNA of Cas13e exemplifies this transition. Its crRNA not only fulfills the traditional role of an antitoxin by inhibiting the non-specific nuclease activity of Cas13e but also possesses a novel function that guides the targeted cleavage of exogenous RNAs. The dual characteristics of Cas13e serve as evidence for its position as a transitional form in evolution. As Cas13e evolved into Cas13a, the non-specific RNase activity of apo Cas13e disappeared, marking the completion of the transformation of the TA system into CRISPR. Structural insights into this transition were provided by cryo-electron microscopy analysis of the PbAbiF RNP complex. Each PbAbiF monomer binds to a single AbiFr ncRNA molecule, similar to other Type III TA systems [113,114]. The HEPN domain of AbiF adopts a canonical four-helix fold and assembles into a symmetric dimmer with active-site residues clustered at the dimer interface. Each AbiF monomer binds one molecule of AbiFr ncRNA, which interacts with the protein via its unique secondary structure elements. Key nucleotides such as C32 and U38 within a specific single-stranded bulge of AbiFr directly inhibit the non-specific RNase activity of AbiF, thereby neutralizing its toxicity (Figure 4). These structural features provide the original template for understanding the evolution of the HEPN domain and the circularly permuted arrangement of the four-helix fold in Cas13a [115]. Evolutionary studies of Cas13a reveal a sophisticated adaptive process. Starting from a simple TA system, the protein gradually evolved into a complex RNA-targeting immune apparatus through sequential events of gene fusion, structural optimization and functional specialization. This evolutionary journey not only offers a novel perspective for deciphering the origin of CRISPR-Cas systems but also provides a theoretical framework for the rational design of multifunctional RNA regulatory tools.

Similar evolutionary strategies are likely widespread across biological systems. It is therefore imperative to investigate the evolutionary relationships between CRISPR and other defense systems, to characterize the structure and function of additional intermediate forms, and to apply this knowledge to engineer novel gene-editing tools with greater practical value. Simultaneously, the ongoing accumulation of metagenomic data and the improving capability of AI-driven structure prediction provide a powerful foundation for discovering further evolutionary secrets concealed within the protein structural space.

## 5. Applications Based on Associating Toxin–Antitoxin with CRISPR-Cas

Research into the synergistic functions between CRISPR-Cas and toxin–antitoxin systems may catalyse the development of groundbreaking biotechnological applications. This functional coupling may hold promise for solving the increasingly severe global crisis of antibiotic resistance. The utilization of CRISPR-Cas technology to engineer sequence-specific antimicrobial agents constitutes a prominent frontier, including the design of CRISPR RNAs that target antibiotic resistance genes within pathogenic bacteria. These crRNAs direct Cas nucleases to introduce DNA double-strand breaks specifically in the target pathogen, leading to the selective eradication of resistant strains [116,117]. However, this strategy is confronted by a fundamental problem: bacterial populations can evolve resistance to the CRISPR-Cas antimicrobials themselves through mutations in the target sequences or the acquisition of anti-CRISPR proteins, thereby inactivating the system [118,119,120,121]. To solve this evasion mechanism, researchers have drawn inspiration from the CreTA TA module to develop a novel strategy termed ATTACK. The core innovation of the ATTACK platform lies in the synthetic biological coupling of the programmable, targeted DNA cleavage activity of CRISPR-Cas with the conditional “suicide switch” functionality inherent to TA systems, thereby establishing a dual-safeguard, precision antimicrobial system. Taking multidrug-resistant *Acinetobacter baumannii* as an example, the design of the ATTACK system is as follows: a modified CreTA module, in which the repetitive sequences of the CreTA module are replaced with versions that the host Cas protein can recognize, is combined with the pathogen’s type I-F CRISPR-Cas system. Under normal conditions, the system expresses crRNA targeting the gentamicin resistance gene aac3. After the CRISPR-Cas system recognizes and cleaves this gene, it renders the pathogen sensitive to antibiotics again or directly causes its death. When the pathogen inactivates the CRISPR-Cas system through mutation or Acr proteins, the “suicide switch” of the CreTA module is activated. Due to the removal of CRISPR regulation, the toxin CreT is highly expressed, killing those strains that attempt to escape CRISPR attack by mechanisms such as interfering with protein synthesis.

Experimental data indicate that the standalone CRISPR antimicrobial demonstrated a bactericidal efficiency of 97.84% and a resistance elimination rate of 91.39% against clinical multidrug-resistant strains. In contrast, the ATTACK strategy achieved a significantly superior performance, with a bactericidal efficiency of 98.96% and a resistance elimination rate of 99.64%, markedly outperforming the single CRISPR-based antimicrobial approach. The ATTACK strategy has fundamental advantages that lie in substantially reducing the escape rate: through a dual-killing mechanism, it greatly raises the genetic threshold for pathogens to develop complete resistance. It also exhibits extremely high specificity, combining the sequence-specific targeting of CRISPR with the conditional logic of toxin expression, enabling precise elimination of specific pathogens and avoiding the damage to commensal microbiota caused by wide-spectrum antibiotic [122,123]. Additionally, this strategy, as a programmable platform, can flexibly target various drug-resistant pathogens by replacing crRNA and adapting to different TA modules (Table 3). The ATTACK strategy represents an advanced technology concept in antimicrobial therapy and even broader applications in synthetic biology. It demonstrated a shift from using single, powerful tools toward designing complex regulatory networks. Rather than simply adopting ready-made natural systems like CRISPR-Cas or TA modules for direct use, this approach involves a profound understanding of their intrinsic mechanisms. By reconfiguring and repurposing the targeting and cleavage capabilities of CRISPR-Cas systems alongside the inhibitory and suicidal functions of TA modules, it creates novel, more powerful systems that do not exist in nature. In the future, research focusing on the mechanisms and principles of naturally coupled systems will help us design more intelligent and powerful synthetic biological systems. These systems can be applied not only in antimicrobial therapy but also in various fields such as environmental microbiome regulation and enhancing the stability of genetic circuits.

## 6. Conclusions and Prospects

The multi-layered functional coupling and deep evolutionary connections between toxin–antitoxin systems and CRISPR-Cas systems have transformed our understanding of prokaryotic defense networks. The three representative systems systematically elaborated in this article—CreTA, CreR, and CrePA—demonstrate their roles in addressing three core challenges within bacterial cells: the evolutionary stability of CRISPR systems, optimization of bacterial physiological costs, and population immunity. Collectively, they establish that prokaryotic defense systems constitute a highly integrated, internally communicating intelligent network. Evolutionary research further reveals that the relationship between TA systems and CRISPR-Cas systems is likely far more complex than previously imagined. The discovery that key CRISPR components, such as Cas2 and Cas13, originated from TA system toxins illustrates that complex adaptive immune machinery was likely assembled gradually from simple regulatory genetic elements through “functional recruitment.” This evolutionary inheritance not only provides a novel perspective for understanding the origins of natural immune systems but also highlights the central role of non-coding RNA throughout this evolution. The understanding of this synergistic interaction is rapidly translating into powerful technological solutions for real-world problems. The ATTACK strategy stands as a prime example, successfully combining the targeting specificity of CRISPR with the “suicide switch” function of TA systems. It offers an innovative solution to the global antibiotic resistance crisis, characterized by high specificity and a low escape rate.

Looking ahead, although the mechanisms and evolutionary origins of the association between several representative TA systems and CRISPR systems have been elucidated, many details remain unclear, and various unresolved questions await answers. For instance, the CreTA system has so far only been clarified in Type I-B systems. Do similar mechanisms widely exist in other CRISPR-Cas types? Similarly, Beyond the FtsZ-targeting toxin in CrePA, do similar systems exist that couple with other CRISPR types and have different cellular targets? Do other types of TA systems exhibit synergistic interactions with CRISPR-Cas systems? What are the specific molecular steps in the transition from the antitoxin function of ncRNA in AbiF to its role as a guide RNA in Cas13e? Did other CRISPR-Cas systems also evolve from toxin–antitoxin systems? In the ATTACK strategy, is it possible for bacteria to develop resistance to both the CRISPR and CreTA components simultaneously? Introducing ATTACK applies intense selective pressure on bacterial populations. Could this accelerate the evolution of more potent or rare resistance mechanisms?

These unanswered questions in the field are likely to guide future research priorities. Within the vast repertoire of TA and CRISPR-Cas systems, many mechanisms undoubtedly remain to be discovered.

## Figures and Tables

**Table 1:** Characteristics of Cas9, Cas12, and Cas13.

Type	Type II	Type V	Type V
Effector	Cas9	Cas12a	Cas13a
Target Molecule	Double-stranded DNA (dsDNA)	Double-stranded DNA (dsDNA)	Single-stranded RNA (ssRNA)
PAM	5′-NGG	5′TTTV	3′H (PTS)
Cleavage Mechanism	Blunt ends	Sticky ends	Cuts ssRNA
Guide RNA	crRNA + tracrRNA (often fused as sgRNA)	crRNA	crRNA
Applications	Gene knockout, knock-in, transcription regulation	Genome editing, Molecular Diagnostics	RNA Knockdown, Molecular Diagnostics
References	[46,47,48]	[49,52]	[55,56]

**Table 2:** Characteristics of CreTA; CreR and CrePA.

Category	CreTA	CreR	CrePA
Type	RNA Toxin–Antitoxin	crRNA-like RNA	Protein Toxin and RNA Antitoxin
Problem Solved	Prevent the host from losing the CRISPR-Cas system	Optimize CRISPR-Cas expression to avoid resource waste and autoimmunity	Eliminate phages that possess Acr proteins and can escape CRISPR defense
Toxin Property	RNA molecule; inhibits translation by hijacking ribosomes/tRNA ^UCU^	No direct toxin; its “toxicity” arises from insufficient Cas proteins leading to defense failure	Protein toxin; interferes with FtsZ to inhibit cell division
Trigger Condition	Disruption or loss of the CRISPR-Cas system	Changes in intracellular crRNA concentration	Inhibition of CRISPR-Cas function by Acr proteins
References	[6]	[74]	[89]

**Table 3:** Comparison of the ATTACK strategy with traditional broad-spectrum antibiotics and CRISPR antimicrobials.

Category	Wide-Spectrum Antibiotic	CRISPR Antimicrobial	ATTACK Strategy
Mechanism	Attack on key physiological processes without distinction	Precisely target specific gene sequences	Dual Targets: Precision Strike by CRISPR and Backup Clearance by TA System
Specificity	Low, disrupting the symbiotic bacterial community	High, theoretically capable of targeting a single strain	Extremely high, combined with Sequence specificity
Drug resistance	Very serious, with a lot of pressure in making a choice.	Easily evasive	Significantly reduce the escape rate
Advantage	Extensively used and with quick effect	High specificity, programmable	Efficient, precise, and extremely difficult to evade
References	[122,123]	[118,119,120,121]	[7]

## Data Availability

No new data were created or analyzed in this study.

## Abbreviations

The following abbreviations are used in this manuscript:

CRISPR	Clustered Regularly Interspaced Short Palindromic Repeats
TA	Toxin–antitoxin
MDR	Multidrug resistance
ATTACK	Associate toxin–antitoxin and CRISPR-Cas to kill MDR pathogens
crRNA	CRISPR RNA
tracrRNA	Trans-activating crRNA
Acr	Anti-CRISPR
MGEs	Mobile genetic elements
HEPN	Higher Eukaryotes and Prokaryotes Nucleotide-binding
